# Attenuation of lung inflammation and fibrosis in CD69-deficient mice after intratracheal bleomycin

**DOI:** 10.1186/1465-9921-12-131

**Published:** 2011-10-05

**Authors:** Keita Yamauchi, Yoshitoshi Kasuya, Fuminobu Kuroda, Kensuke Tanaka, Junichi Tsuyusaki, Shunsuke Ishizaki, Hirofumi Matsunaga, Chiaki Iwamura, Toshinori Nakayama, Koichiro Tatsumi

**Affiliations:** 1 Department of Respirology, Graduate School of Medicine, Chiba University, Chiba, Japan; 2 Department of Biochemistry and Molecular Pharmacology, Graduate School of Medicine, Chiba University, Chiba, Japan; 3 UBE Industries, Ltd., Ube, Japan; 4 Department of Immunology, Graduate School of Medicine, Chiba University, Chiba, Japan

**Keywords:** cluster of differentiation 69, lung inflammation, pulmonary fibrosis, bleomycin

## Abstract

**Background:**

Cluster of differentiation 69 (CD69), an early activation marker antigen on T and B cells, is also expressed on activated macrophages and neutrophils, suggesting that CD69 may play a role in inflammatory diseases. To determine the effect of CD69 deficiency on bleomycin(BLM)-induced lung injury, we evaluated the inflammatory response following intratracheal BLM administration and the subsequent fibrotic changes in wild type (WT) and CD69-deficient (CD69^-/-^) mice.

**Methods:**

The mice received a single dose of 3 mg/kg body weight of BLM and were sacrificed at 7 or 14 days post-instillation (dpi). Lung inflammation in the acute phase (7 dpi) was investigated by differential cell counts and cytokine array analyses of bronchoalveolar lavage fluid. In addition, lung fibrotic changes were evaluated at 14 dpi by histopathology and collagen assays. We also used reverse transcription polymerase chain reaction to measure the mRNA expression level of transforming growth factor β1 (TGF-β1) in the lungs of BLM-treated mice.

**Results:**

CD69^-/- ^mice exhibited less lung damage than WT mice, as shown by reductions in the following indices: (1) loss of body weight, (2) wet/dry ratio of lung, (3) cytokine levels in BALF, (4) histological evidence of lung injury, (5) lung collagen deposition, and (6) TGF-β1 mRNA expression in the lung.

**Conclusion:**

The present study clearly demonstrates that CD69 plays an important role in the progression of lung injury induced by BLM.

## Background

Idiopathic pulmonary fibrosis (IPF) is a chronic interstitial pneumonia of unknown causes and has poor prognosis [1,2]. Patients with IPF could be treated with steroids or immunosuppressants to ameliorate the inflammation that occurs early in the course of the disease, but these drugs do not improve their survival [3]. Hence, the discovery of a target that could be useful in the therapeutic intervention of IPF is desirable.

Bleomycins (BLMs) are a family of glycopeptide antibiotics [4] with potent anti-tumor activity against a wide range of lymphomas, head and neck cancers, and germ-cell tumors [5]. However, the therapeutic efficacy of BLM is limited by the development of pulmonary fibrosis in patients using it [6,7]. BLM-induced pulmonary fibrosis in mice is the most common experimental model of human IPF. In this model, intratracheal administration of BLM induces acute alveolitis and interstitial inflammation, which are characterized by the recruitment of leukocytes within 1 week [8] and pulmonary edema. Subsequently, during the second week, fibrotic responses, such as fibroblast proliferation and synthesis of extracellular matrix, occur [9]. Various types of cells, including macrophages and neutrophils have been the immune cells primarily implicated as playing potential roles in the development of pulmonary fibrosis [10].

Cluster of differentiation 69 (CD69) is a C-type lectin expressed as a disulfide-linked homodimeric membrane protein [11]. The CD69 gene is located within the natural killer (NK) gene complex on mouse chromosome 6 and human chromosome 12 [12,13]. CD69 was initially detected on the surface of activated lymphocytes and is known as a very early activation marker antigen [14-16]. However, CD69 expression is not restricted to these cells, since activated macrophages, neutrophils, and eosinophils can also express CD69 [17-19]. Moreover, antibody crosslinking of CD69 induces several cellular responses, including nitric oxide (NO) production and release of tumor necrosis factor α (TNF-α) in murine macrophages [17], NO production in human monocytes [20], neutrophil degranulation [18], T cell proliferation and production of TNF-α [21,22], and NK cell cytotoxicity [23]. These facts indicate that CD69 exerts a potential proinflammatory function and may be involved in the pathogenesis of inflammatory diseases such as pulmonary fibrosis. To determine the effects of CD69 deficiency on BLM-induced lung injury, we evaluated the inflammatory response to intratracheal BLM administration and the subsequent fibrotic changes in wild-type (WT) and CD69-deficient (CD69^-/-^) mice.

## Materials and methods

### Mice

Eight-week-old male C57BL/6J mice were purchased from Clea Japan (Tokyo, Japan). CD69^-/- ^mice [24] were backcrossed with C57BL/6J 10 times. Male CD69^-/- ^and WT mice (8-10 weeks) were used in this study. All mice used in this study were bred in the Animal Resource Facility at Chiba University under pathogen-free conditions and cared for according to the animal care guidelines of Chiba University.

### Induction of lung injury by bleomycin

Prior to experimentation, mice were weighed and anaesthetized with an intraperitoneal injection of tribromoethanol. Subsequently, the animals were given a single intratracheal injection of BLM hydrochloride (3 mg⋅kg^-1^; Nippon Kayaku, Tokyo, Japan) dissolved in phosphate-buffered saline (PBS) by using a Microsprayer^® ^atomizer (PennCentury, Philadelphia, PA). Control mice received a sham treatment of PBS.

### Measurement of fluid content in lung

The right lung was carefully excised, and then its wet weight was measured. Subsequently, the lung was dried for 24 h at 60°C, and then its dry weight was measured. The ratio between wet and dry lung weight is a measure of edema formation.

### Collection of bronchoalveolar lavage fluid

Seven days after BLM administration, mice were anesthetized with pentobarbital (Schering-Plough, Kenilworth, NJ) and sacrificed. The trachea was exposed and lavaged 3 times with 1 mL of PBS by using a 20-gauge catheter. The lavage fluids were pooled and then centrifuged at 300 × *g *for 5 min at 4°C. The resulting supernatants were stored at -80°C for chemokine and cytokine measurements. The pellets were resuspended in PBS to determine the total and differential cell counts of the bronchoalveolar lavage fluid (BALF). The total cell count was measured by using a hemocytometer. The differential cell count was determined by manually counting 200 cells per mouse that were stained with Diff-Quick (Sysmex Corporation, Kobe, Japan) and fixed on glass slides.

### Measurement of cytokine levels

The level of cytokines in the BALF was measured by a RayBio mouse inflammation antibody array 1 (RayBiotech, Norcross, GA). This assay employs a qualitative westernblot (WB) screening technique that can detect 80 cytokines. We precisely followed the manufacturer's protocol. Briefly, the membranes were placed in an 8-well tissue culture tray and incubated with blocking buffer at room temperature for 30 min. One milliliter of BALF sample was added to each membrane and incubated for 2 h. After the samples were removed and washed, the membranes were incubated with the biotin-conjugated antibodies specific for cytokines overnight at 4°C. After washing, the membranes were incubated with 1:1,000 diluted horseradish peroxidase-conjugated streptoavidin for 2 h at room temperature. Next, the membranes were incubated with chemiluminescent detection buffer, wrapped in plastic wrap, and exposed to radiographic film (Kodak X-Omat; Kodak; Rochester, NY) for 40 s. The visualized signals on the developed film were quantified by an image-processing and analysis program (Fuji Image Gauge software version 3.0; Fujifilm, Tokyo, Japan).

### Histological examination

Lung biopsies were taken at 14 dpi with BLM or PBS. Lung tissues were inflated and fixed in 4% paraformaldehyde, embedded in paraffin, and cut into 8-μm-thick sections. Sections were subjected to hematoxylin and eosin (H-E) or Masson trichrome stain (Sigma-Aldrich, St. Louis) according to the manufacturer's instructions. The severity of the fibrosis was semi-quantitatively assessed according to the method proposed by Ashcroft and coworkers [25]. Briefly, the grade of lung fibrosis was scored on a scale from 0 to 8 by examining random sections at 100 × magnification. The general scoring criteria were as follows: grade 0, normal lung; grade 1, minimal fibrous thickening of the alveolar or bronchiolar walls; grade 3, moderate thickening of the walls without obvious damage to lung architecture; grade 5, significant fibrosis with obvious damage to lung structure and formation of fibrous bands or small fibrous masses; grade 7, severe distortion of lung structure and large fibrous masses; grade 8, total fibrous obliteration of the field. The Ashcroft score of each lung section was reported as the mean score of at least 20 microscopic fields.

### Collagen assay

Desiccated caudal lobes from the right lung 14 d after BLM administration were homogenized with 0.1 mg/mL pepsin (Wako chemicals, Osaka, Japan) in 0.5 mol/L acetic acid and incubated for 24 h at 4°C while stirring constantly. Subsequently, the samples were centrifuged at 10000 × *g *for 5 min at 4°C. The total lung collagen content of the supernatant was measured by using the Sircol Collagen Assay kit (Biocolor, Belfast, Northern Ireland).

### Expression of TGF-ß1 in bleomycin-treated lung

Mice were sacrificed at 7 dpi, and the lung was dissected out. The RNA of the lung was isolated using ISOGEN (Wako chemicals) according to the manufacturer's instructions. Single-stranded cDNA was synthesized from prepared RNA (1 μg) with Moloney murine leukemia virus reverse transcriptase (Invitrogen, Carlsbad, CA) using an oligo(dT) primer (Invitrogen) in a total volume of 20 μL. The resultant cDNA sample (1 μL) was subjected to PCR for the amplification of mouse TGFß-1 cDNA using specific primers (sense primer, 5'-CAACAACGCCATCTATGAGA-3'; antisense primer, 5'-TATTCCGTCTCCTTGGTTC-3'). As an internal control, mouse glyceraldehyde-3-phosphate dehydrogenase (GAPDH) cDNA was amplified using specific primers (sense primer, 5'-GACCACAGTCCATGACATCACT'-3; antisense primer, 5'-TCCACCACCCTGTTGCTGTAG-3'). The settings of the thermal cycler were 30 cycles of 45 s at 94°C, 1 min at 52°C, and 1 min at 72°C for mouse TGFß-1 and 25 cycles of 40 s at 94°C, 1 min at 60°C, and 1 min at 72°C for mouse GAPDH. The amplified products were separated on a 1.2% agarose gel and visualized with ethidium bromide staining under UV radiation. Specific amplification of the expected sizes (mouse TGFß-1, 295 bp; mouse GAPDH, 452 bp) was observed.

### Immunohistochemical analysis

The lung fixed in 4% paraformaldehyde/0.1M sodium phosphate buffer (pH 7.4) was embedded in OCT (SAKURA Finetek, Tokyo) and cut into 10-μm-thick sections, which were placed on poly-L lysine-coated slides. The sections were subjected to double staining with hamster anti-CD69 monoclonal antibody (Biolegend, San Diego, CA) in combination with rabbit anti-Iba1 polyclonal antibody (Wako chemicals, Tokyo) followed by a reaction with Alexa Fluor 594-conjugated anti-hamster antibody or Alexa Fluor 488-conjugated anti-rabbit antibody. The sections were simultaneously stained with 4',6-diamino-2-phenylindole (DAPI). To confirm the precise localization of Iba1-positive cells, the sections were stained with hamster anti-podoplanin/gp36 monoclonal antibody, which detects alveolar epithelial cells and thereby clearly visualizes the alveolar structure.

### Statistical analysis

The Student's t-test or analysis of variance (ANOVA) with the Newman-Keuls test was used to determine whether results were statistically significant (P < 0.05). All statistical analyses were performed with GraphPad PRISM software (Version 5.0 for Windows; GraphPad, San Diego, CA).

## Results

### Body weight change

**Figure 1:**
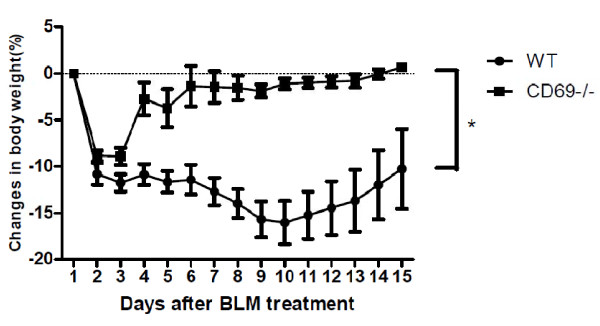
**Effect of bleomycin treatment on body weight in wild-type and CD69-deficient mice**. Time course of changes in body weight after bleomycin (BLM) treatment in wild-type (WT) (*n *= 8) and cluster of differentiation 69 (CD69)-deficient (CD69^-/-^) mice (*n *= 6). Results are expressed as the mean (SEM), *P < 0.05.

To determine the biological significance of CD69 deficiency after acute lung injury, we tracked weight changes following BLM exposure. WT mice showed typical and persistent body weight loss after BLM exposure. In contrast, the CD69^-/- ^mice transiently lost body weight after BLM exposure but underwent steady weight gain thereafter. Between the two groups, a marked difference was observed (Figure 1). In mice injected with PBS, the mice body showed daily weight gain without any loss (data not shown).

### Differential cell counts in bronchoalveolar lavage fluid and lung edema

**Figure 2:**
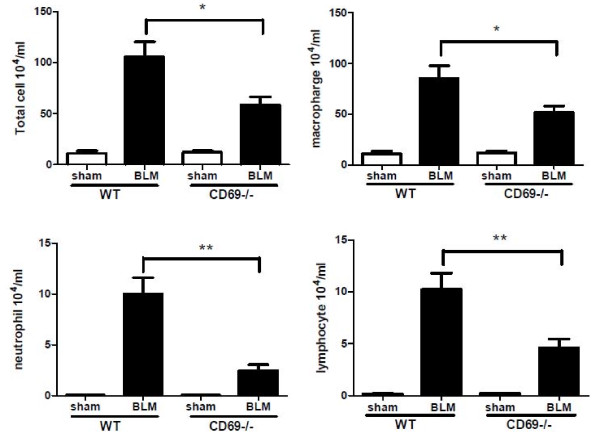
**Effect of bleomycin treatment on differential cell counts in wild-type and CD69-deficient mice**. Differential cell counts in bronchoalveolar fluid (BALF) were determined 7 d after the instillation of BLM or phosphate-buffered saline (sham treatment). Results are expressed as the mean (SEM) (*n *= 6-8 BLM-treated mice, *n *= 3 sham-treated mice). *P < 0.05, **P < 0.01.

**Figure 3:**
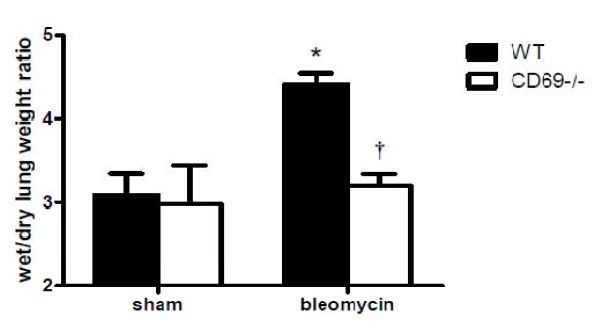
**Effect of bleomycin treatment on lung fluid content in wild-type and CD69-deficient mice**. Ratio of wet/dry lung weight 7 d after the instillation of BLM or PBS (sham treatment). Results are expressed as the mean (SEM) (*n *= 8 WT and 6 CD69^-/- ^mice). The ratio between wet and dry lung weight is a measure of edema formation. *P < 0.01 vs. sham-treated mice. †*P *< 0.01 vs. WT mice.

To determine whether the CD69 deficiency affected the BLM-induced infiltration of inflammatory cells into the airways and parenchyma, we differentially counted the inflammatory cells in BALF at 7 dpi. As shown in Figure 2, the numbers of total inflammatory cells, macrophages, neutrophils, and lymphocytes in the BALF were significantly elevated in the BLM-injected mice compared to those in the PBS-injected mice (sham). Moreover, the increase in these cell populations in the BLM-induced mice was significantly attenuated in the CD69^-/- ^mice. Intratracheally BLM-treated mice showed an inflammatory response characterized by the accumulation of water in the lungs, indicative of tissue edema. CD69 deficiency significantly reduced the lung fluid content resulting from BLM-treatment (Figure 3).

### Inflammatory cytokine levels in bronchoalveolar lavage fluid

**Figure 4:**
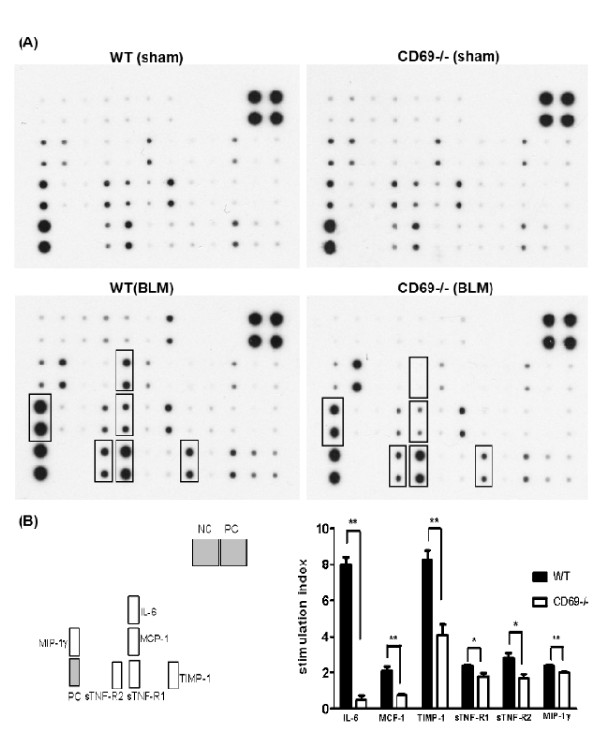
**Effect of bleomycin treatment on cytokine expression in wild-type and CD69-deficient mice**. (**A**) Cytokine array analyses of BALF 7 d after the instillation of BLM or PBS (sham treatment) in WT or CD69^-/- ^mice. Cytokines with increased expression levels are boxed. (**B**) Expression levels of these cytokines in WT (black bars) and CD69^-/- ^(white bars) mice. Each expression level was normalized by that of the positive control. The stimulation index is the ratio of the expression level of a cytokine in BLM-treated mice to that in sham-treated mice. Results are expressed as the mean (SEM) (*n *= 4 mice per group). *P < 0.05, **P < 0.01.

To evaluate whether CD69 deficiency affected the inflammatory responses induced by BLM, we comprehensively investigated the differences in the expression of cytokines involving chemokines in the BALF. As shown in Figure 4A, the expression of several cytokines and chemokines in the BALF from WT and CD69^-/- ^mice was induced by BLM. We focused on IL-6, MCP-1, TIMP-1, sTNF-R1, sTNF-R2, and MIP-1γ (Figure 4A &4B). These cytokines and chemokines were clearly induced by BLM in WT mice but less so in CD69^-/- ^mice. Thus, the decreased expression of these factors may be closely related to the mechanisms underlying the attenuation of symptoms in CD69^-/- ^mice, including the reduction in leukocytic infiltration and edema. An analysis by densitometer revealed that the expression levels of these cytokines and chemokines in BLM-treated CD69^-/- ^mice were significantly lower than those in WT mice (Figure 4B).

### Histological and biochemical changes in lung

**Figure 5:**
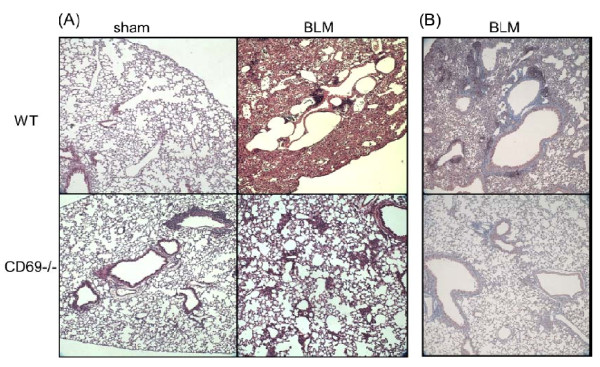
**Effect of bleomycin on the lung architecture in wild-type and CD69-deficient mice**. Comparison of the lung architecture in WT and CD69^-/- ^mice after instillation of BLM or PBS (sham treatment), as shown by hematoxylin-eosin (**A**) and Masson's trichrome (**B**) staining of representative tissue sections.

**Figure 6:**
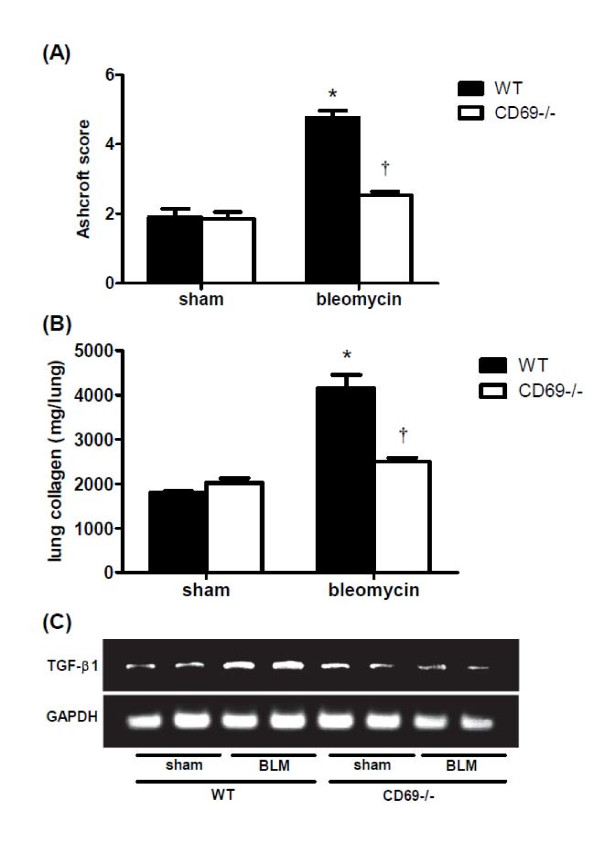
**Effect of bleomycin on lung fibrotic and biochemical changes in wild-type and CD69-deficient mice**. (**A**) Ashcroft scores, which are a semi-quantitative measure of lung fibrotic changes, were determined 14 d after the instillation of BLM or PBS (sham treatment). Please see the Methods section for an explanation of the scoring criteria. Results are expressed as the mean (SEM) (*n *= 4 mice per group). *P < 0.01 vs. sham-treated mice. †*P *< 0.01 vs. WT mice. (**B**) The lung collagen content was measured 14 d after the instillation of BLM or PBS (sham treatment). Results are expressed as mean (SEM) (*n *= 6-8 BLM-treated mice, *n *= 3 sham-treated mice). *P < 0.01 vs. sham-treated mice. †*P *< 0.01 vs. WT mice. (**C**) The mRNA expression level of TGF-β1 in the lung was measured 7 d after the instillation of BLM or PBS in WT or CD69KO mice.

For investigating the effects of CD69 deficiency on BLM-induced lung fibrosis, the histopathological changes in the lung were evaluated at 14 dpi. Representative microscopic findings following H-E or Masson's trichrome staining of the lung sections are shown in Figure 5. The lung architecture was nearly normal between the two genotypes injected with PBS. However, the WT lung tissue exposed to BLM showed a strong accumulation of inflammatory cells, thickening of the alveolar walls, and fibrotic lesions. Although these findings were also observed in the CD69^-/- ^mice, the extent and intensity were much less than those in the WT mice. The severity of the fibrosis was also assessed by Ashcroft scoring. This assessment confirmed that the severity of fibrosis was significantly reduced in BLM-treated CD69^-/- ^mice relative to that in the correspondingly injured WT mice (Figure 6A). In accordance with the results of the Ashcroft scoring, collagen deposition was markedly developed in the lungs of BLM-treated WT mice at 14 dpi compared to that in the sham group. Moreover, the increased collagen contents induced by BLM were significantly attenuated in CD69^-/- ^mice (Figure 6B). TGF-β1 mRNA expression in the lung tissue was measured by RT-PCR at 7 dpi. The BLM-induced expression of TGF-ß1 was strongly reduced in CD69^-/- ^mice relative to that in WT mice (Figure 6C).

### Predominant localization of CD69 in lung

**Figure 7:**
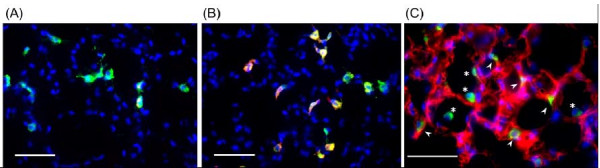
**Expression of CD69 on macrophages in the lung**. The lung from WT mice at 2 dpi with PBS (sham treatment) (**A**) or BLM (**B**) were subjected to immunohistochemical staining with an anti-CD69 antibody and an anti-Iba1 antibody, followed by a reaction with Alexa Fluor 594-conjugated and Alexa Fluor 488-conjugated secondary antibodies, respectively. The control lung from WT mice was subjected to immunohistochemical staining with an anti-gp36 antibody and an anti-Iba1 antibody, followed by a reaction with Alexa Fluor 594-conjugated and Alexa Fluor 488-conjugated secondary antibodies, respectively (**C**). Each arrowhead points to an interstitial macrophage. Each asterisk indicates an alveolar macrophage. All sections were co-stained with DAPI. Each bar represents 50 μm.

As shown in Figure 7A, Iba1-positive macrophages were observed in the lungs from WT PBS-injected mice but rarely exhibited CD69-like immunoreactivity. On the other hand, Iba1^+^/CD69^+ ^macrophages were clearly observed in the lung from WT BLM-treated mice at 2 dpi (Figure 7B), indicating that the expression of CD69 was induced in the macrophages exposed to BLM. In addition, Iba1 recognized alveolar and interstitial macrophages, suggesting that BLM induced CD69 expression in the two types of macrophages (Figure 7C). At 2 dpi after BLM injection, T cells and neutrophils were barely detected as infiltrating cells in the lung (data not shown).

## Discussion

The ability of CD69 to function as a signal transducing molecule in various types of cells, together with its upregulation in certain inflammatory diseases, suggests a possible pathogenic role for CD69 [26]. Indeed, CD69 is persistently expressed in the infiltrates of leukocytes produced during the course of chronic inflammatory diseases such as chronic hepatitis [27] and rheumatoid arthritis [28]. Recently, Miki *et al. *demonstrated that CD69 expressed on CD4-positive T cells plays a critical role in the development of allergen-induced eosinophilic inflammation [29]. These findings led us to consider that the gene disruption of CD69 could affect the pathogenesis of pulmonary inflammatory diseases. To address to this notion, we investigated the development of BLM-induced lung injury in CD69^-/- ^mice.

The importance of a profibrotic inflammatory process in the pathogenesis of pulmonary fibrosis has been suggested in a number of studies that have found that lung injury leads to an inflammatory reaction characterized by the production of inflammatory cytokines and the recruitment of leukocytes [30]. In this study, we showed that inflammatory responses such as an accumulation of inflammatory cells in the BALF and lung edema occur after instillation with BLM. These parameters were significantly reduced in CD69^-/- ^mice compared with WT mice (Figures 2 &3). The differences in the inflammatory parameters between the CD69^-/- ^and WT mice were consistent with the differences in the body weight profiles reflecting a pathological state in the mice (Figure 1). These results suggest that CD69^-/- ^mice are more resistant to BLM-induced lung inflammation than WT ones. This conclusion was supported by the WB array analysis for cytokines/chemokines. The expression of BLM-induced cytokines/chemokines (e.g., MCP-1, IL-6, TIMP-1) in the WT mice was significantly reduced in CD69^-/- ^mice (Figure 4). Likewise, these cytokines/chemokines have been reported to play an important role in the pathogenesis of pulmonary fibrosis [31-34]. Thus, the reduced expression of these cytokines/chemokines appears to be at least partly responsible for the suppression of the BLM-induced lung inflammation that leads to fibrosis in CD69^-/- ^mice.

TGF-β1 plays a critical role in the pathogenesis of lung fibrosis through the stimulation of collagen and fibronectin production in fibroblasts [35]. Likewise, it is well established that the initial elevation of pro-inflammatory cytokines leads to an increase in the expression of pro-fibrotic markers involving TGF-β1. This increased expression appears to occur in the following manner: 1) MCP-1 may act as a profibrotic mediator by promoting fibroblast procollagen gene expression through the up-regulation of TGF-β1 [36]; and 2) TNF signaling through sTNF-receptors contributes to the regulation of TGF-β1 expression during BLM-induced lung fibrosis, as mice lacking sTNF-receptors have been shown to be resistant to BLM-induced lung fibrosis [30]. These facts strongly suggest the possibility that the expression of cytokines/chemokines involving MCP-1 and sTNF-receptors in the lung can affect the extent of TGF-β1 expression and the fibrotic tissue profiles. As expected, BLM-induced expression of TGF-β1 mRNA prior to fibrosis was suppressed in the lungs of CD69^-/- ^mice relative to that in WT mice (Figure 6C). Furthermore, lung fibrosis was markedly attenuated in the CD69^-/- ^mice (Figures. 5, 6A &6B).

By immunohistochemical analysis, CD69 was predominantly expressed in macrophages after BLM administration in WT mice at an earlier stage (Figure 7). At this time point, the infiltration of T cells and neutrophils was not observed. These results suggest that CD69 on macrophages may play an important role in the initial step of BLM-induced lung injury. Macrophages are thought to play a pivotal role in the pathogenesis of pulmonary fibrosis. Activated macrophages secrete a variety of enzymes, complement components, cytokines, and other mediators of inflammatory and fibroblast cell function [37]. It has been also reported for *in vitro *and *in vivo *studies that alveolar macrophages release proinflammatory cytokines after BLM administration [38]. Furthermore, it is thought that alveolar macrophages, following stimulation by BLM-induced injury, secrete a large quantity of TGF-β1 and thereby induce the lung fibroblasts in the alveolar interstitium to synthesize collagen, resulting in pulmonary fibrosis [39,40]. These suggest that BLM-activated macrophages may function as one of major sources of chemical mediators in the pulmonary inflammation/fibrosis loop. Indeed, the majority of inflammatory cells recovered by BAL were macrophages, which were of an order of magnitude higher in number than those of neutrophils and lymphocytes (Figure 2).

An important question is the role of CD69 on macrophages in the induction of IPF. Previous studies have reported the ability of the CD69 antigen to act as a potent trigger of murine macrophage activation [17]. The stimulation of macrophages with anti-CD69 mAb has been shown to induce both NO production and TNF-α release. Likewise, it has been found that CD69 cross-linking induces TGF-β1 production in macrophages as well as in T cells and NK cells [41,42]. We also confirmed a clear difference between macrophages from WT mice and those from CD69^-/- ^mice in the secretion of LPS-induced cytokines/chemokines (unpublished data). Hence, it is indisputable that CD69 can participate in the activation and regulation of macrophages in the inflammatory process. Although the signaling loop of CD69/TGF-β in activated macrophages may contribute to IPF in a direct manner, it is of interest as to whether BLM-associated tissue injury induces the expression of the putative CD69 ligand on certain cells, such as epithelial cells, especially from the standpoint of epithelial mesenchymal transition (EMT). Further study will be necessary to determine the precise molecular mechanisms of CD69-mediated development in IPF.

## Conclusion

We have shown for the first time that CD69 plays a key role in promoting inflammation and fibrosis in the BLM-injured lung. The present study suggests that CD69 may be a potentially useful target in the therapeutic intervention of IPF.

## Competing interests

The authors declare that they have no competing interests.

## Authors' contributions

KY carried out experimental work, data analysis and manuscript drafting. JT, SI and HM assisted in animal experiments, participated in study design. K. Tanaka carried out RT-PCR and Immunohistochemistry. HK participated in study design and helped to draft the manuscript. CI and TN participated in study coordination. YK and K. Tatsumi conceptualized of the study and supervised this project. All authors have read and approved the final manuscript.
